# Impact of extended lung protection during mechanical ventilation on lung recovery in patients with COVID-19 ARDS: a phase II randomized controlled trial

**DOI:** 10.1186/s13613-024-01297-z

**Published:** 2024-06-08

**Authors:** Eduardo L. V. Costa, Glasiele C. Alcala, Mauro R. Tucci, Ewan Goligher, Caio C. Morais, Jose Dianti, Miyuki A. P. Nakamura, Larissa B. Oliveira, Sérgio M. Pereira, Carlos Toufen, Carmen S. V. Barbas, Carlos R. R. Carvalho, Marcelo B. P. Amato

**Affiliations:** 1 Laboratório de Pneumologia LIM-09, Faculdade de Medicina, Hospital das Clinicas HCFMUSP, Universidade de Sao Paulo, 455 Dr Arnaldo Ave, Room 2144, São Paulo, SP Brazil; 2 Research and Education Institute, Hospital Sírio-Libanes, Sao Paulo, Brazil; 3 Divisao de Pneumologia, Faculdade de Medicina, Instituto do Coracao, Hospital das Clinicas HCFMUSP, Universidade de Sao Paulo, São Paulo, SP Brasil; 4 Interdepartmental Division of Critical Care Medicine, University of Toronto, Toronto, Canada; 5 Keenan Research Centre, Li Ka Shing Knowledge Institute, St. Michael’s Hospital, Toronto, Canada; 6 Toronto General Hospital Research Institute, Toronto, Canada; 7 Departamento de Fisioterapia, Universidade Federal de Pernambuco, Recife, PE Brazil; 8 Adult ICU Albert Einstein Hospital, São Paulo, Brazil

**Keywords:** Positive-pressure ventilation, Lung injury, Acute respiratory distress syndrome, Electrical impedance tomograph

## Abstract

**Background:**

Protective ventilation seems crucial during early Acute Respiratory Distress Syndrome (ARDS), but the optimal duration of lung protection remains undefined. High driving pressures (ΔP) and excessive patient ventilatory drive may hinder lung recovery, resulting in self-inflicted lung injury. The hidden nature of the ΔP generated by patient effort complicates the situation further. Our study aimed to assess the feasibility of an extended lung protection strategy that includes a stepwise protocol to control the patient ventilatory drive, assessing its impact on lung recovery.

**Methods:**

We conducted a single-center randomized study on patients with moderate/severe COVID-19-ARDS with low respiratory system compliance (C_RS_ < 0.6 (mL/Kg)/cmH_2_O). The intervention group received a ventilation strategy guided by Electrical Impedance Tomography aimed at minimizing ΔP and patient ventilatory drive. The control group received the ARDSNet low-PEEP strategy. The primary outcome was the modified lung injury score (_m_LIS), a composite measure that integrated daily measurements of C_RS_, along with oxygen requirements, oxygenation, and X-rays up to day 28. The _m_LIS score was also hierarchically adjusted for survival and extubation rates.

**Results:**

The study ended prematurely after three consecutive months without patient enrollment, attributed to the pandemic subsiding. The intention-to-treat analysis included 76 patients, with 37 randomized to the intervention group. The average _m_LIS score up to 28 days was not different between groups (P = 0.95, primary outcome). However, the intervention group showed a faster improvement in the _m_LIS (1.4 vs. 7.2 days to reach 63% of maximum improvement; P < 0.001), driven by oxygenation and sustained improvement of X-ray (P = 0.001). The intervention group demonstrated a sustained increase in C_RS_ up to day 28 (P = 0.009) and also experienced a shorter time from randomization to room-air breathing (P = 0.02). Survival at 28 days and time until liberation from the ventilator were not different between groups.

**Conclusions:**

The implementation of an individualized PEEP strategy alongside extended lung protection appears viable. Promising secondary outcomes suggested a faster lung recovery, endorsing further examination of this strategy in a larger trial.

*Clinical trial registration* This trial was registered with ClinicalTrials.gov (number NCT04497454) on August 04, 2020.

**Supplementary Information:**

The online version contains supplementary material available at 10.1186/s13613-024-01297-z.

## Background

Acute Respiratory Distress Syndrome (ARDS) affects approximately one-third of mechanically ventilated patients with a high mortality rate [1]. A significant factor to negative clinical outcomes is ventilator-induced lung injury (VILI), which has been largely avoided using protective ventilatory protocols developed over the past few decades [2–7]. Optimal duration of this lung protection, however, remains an unexplored aspect of mechanical ventilatory strategies.

The importance of protective settings during the early stages of ARDS is widely acknowledged [2–7]. Accordingly, many protocols have focused on the early hours and days of mechanical ventilation. The ARDSNet protocol [2], for example, recommends maintaining low tidal volumes for at least 12 h before loosening control if gas exchange improves sufficiently: after transition to pressure support, strict control over tidal volumes is no longer recommended. There is, however, uncertainty about whether this short-term application of these protective measures during ARDS is sufficient to prevent VILI, or if extended protection throughout the mechanical ventilation period is necessary. Concerns exist that inappropriately high patient ventilatory drive can result in high total ΔP leading to self-inflicted lung injury [8–11] and impeding a safe transition from injury to recovery. This phenomenon is especially elusive because of the occult nature of the ΔP generated by the patients’ own efforts.

Both insufficient and excessive patients’ respiratory efforts during spontaneous breathing can pose risks to the lungs and diaphragm [12–14]. This fragile balance underscores the significant challenge of ensuring safe spontaneous breathing; achieving an optimal level of respiratory effort is paramount to avoid these harmful extremes. More specifically, too low patients’ efforts might lead to ventilator-induced diaphragmatic dysfunction (VIDD) [13–15], a harmful side effect that arises from excessive use of ventilatory support, too deep sedation, and muscle paralysis. Conversely, excessive respiratory muscle efforts can in turn cause harmful lung stress, strain, and also diaphragmatic myotrauma [13, 16].

The necessity to balance these factors led to the proposition of a lung and diaphragm-protective (LDP) strategy [12]. This approach sought to create a balance between limiting ventilator-induced harm to both diaphragm and lungs during the whole recovery period of the lungs. In this prior uncontrolled study, the relative contributions of different measures to avoid both too low and too high patient effort were assessed. These measures included titration of sedation and analgesia, pharmacological control of hyperactive delirium, PEEP adjustments, and partial neuromuscular blockade when necessary. They found that respiratory effort was frequently absent or excessive in patients with respiratory failure. Their systematic approach achieved lung- and diaphragm-protective targets in most patients. We decided to test the feasibility and efficacy of a similar strategy in a phase-2 randomized trial.

We hypothesized that a ventilatory strategy that allows early spontaneous breathing but limits total ΔP until extubation could be superior to a standard low tidal volume ventilation, presenting better evolution of a modified lung injury score (_m_LIS) [17] and faster recovery of normal lung function up to day 28 in patients with COVID-19-ARDS.

## Methods

We performed this prospective, randomized, open-label trial in the Respiratory Intensive Care Unit (ICU) Incor/HC-FMUSP, University of Sao Paulo, Sao Paulo, Brazil. The protocol was approved by the institutional review board (approval number 4.001.231). This trial was registered with ClinicalTrials.gov (number NCT04497454) on August 04, 2020. Informed consent was waived due to visiting constraints imposed by the pandemic. Patients under mechanical ventilation with a diagnosis of ARDS [18] < 24 h caused by SARS-COV2 infection, exhibiting respiratory system compliance ≤ 0.6 (mL/Kg of predicted body weight or PBW)/cmH_2_O and an PaO_2_:F_I_O_2_ ≤ 200 mmHg, were included. These inclusion criteria were defined to predictively enrich the study cohort, selectively identifying patients with a higher likelihood of benefiting from the interventions. According to these entry criteria, a patient with tidal volume of 6 mL/Kg would require a driving pressure of ≥ 10 cmH_2_O. The main exclusion criteria were (full list in the Additional file 1) age < 18 years, history of chronic and disabling respiratory disease, impossibility of monitoring with EIT, hemodynamic instability, intracranial hypertension, pregnancy, and refusal of the attending physician to include the patient in the study.

### Pre-randomization monitoring and procedures

All patients were monitored with EIT before randomization and underwent a 30 min phase of ventilator settings according to the ARDSNet protocol [2]. Arterial blood gases were collected at the end of this period.

All patients were then submitted to a lung recruitment maneuver followed by a decremental PEEP titration guided by EIT. In pressure-controlled ventilation, PEEP was gradually increased up to 30 (BMI < 35 kg/m^2^) or 35 cmH_2_O (BMI ≥ 35 kg/m^2^) with a ΔP of 15 cmH_2_O, I: E 1:1, respiratory rate between 20 and 30 breaths/min and inspired fraction of oxygen (F_I_O_2_) of 1. These parameters were maintained for one minute, PEEP was then reduced to 24 cmH_2_O, and the mode was switched to volume-controlled ventilation with a tidal volume of 5 mL/Kg PBW (to minimize tidal recruitment and also to avoid high plateau pressures at the highest PEEP levels), respiratory rate of 20 breaths/min, F_I_O_2_ of 1 and I:E 1:2. With the PEEP titration tool (Enlight^®^1800 or 2100, Timpel medical, Brazil), collapse and overdistension were estimated at each 2 cmH_2_O decremental PEEP step every 30 s until a minimum PEEP level of 4 cmH_2_O. The PEEP associated with equal amounts of collapse and overdistension was defined as EIT_PEEP_.

### Randomization

Eligible patients were randomized in a 1:1 ratio to the extended protection group or to the control group (low PEEP-F_I_O_2_ table ARDSNet). Allocation concealment was achieved by use of an online platform available 24 h a day. The random allocation list was generated in variable blocks of two and four by an investigator not involved in patient inclusion.

### Ventilation protocols

In both groups, the target arterial oxygenation was 55–80 mmHg or saturation 90–95%. Rescue measures for refractory hypoxemia (e.g. inhaled nitric oxide or extracorporeal membrane oxygenation) were allowed in both arms at the discretion of the attending physician. Prone positioning was encouraged in both arms when PaO_2_:F_I_O_2_ was < 150 mmHg.

### ARDSNet protocol

In the ARDSnet arm (control group), patients were ventilated according to the low-PEEP, low-tidal-volume strategy [2]. Patients were placed on volume-controlled mode with the tidal volume set to 6 mL/Kg PBW, plateau pressure ≤ 30 cmH_2_O, and respiratory rate (RR) ≤ 35 breaths/min. The target pH was 7.30–7.45. If pH was between 7.15 and 7.30, RR was increased to reach a pH > 7.30 or PaCO_2_ < 25 mmHg (max = 35 breaths/min). If pH was < 7.15 and RR was at the maximum value allowed, tidal volume was increased even if plateau pressure exceeded 30 cmH_2_O. In this scenario, sodium bicarbonate infusion was also considered. PEEP and F_I_O_2_ were set according to the PEEP-F_I_O_2_ table. At least *12 h* after the start of the protocol, every patient was screened for the commencement of the weaning phase. If the patient reached an F_I_O_2_ ≤ 40%, he or she could be transitioned to pressure support mode with pressure support.

After the initial 12 h of lung protection, patients could be ventilated in volume assist-control mode. During this assisted phase, tidal volumes were controlled with the same targets of the controlled phase. In case of excessive flow starvation (airway pressures during inspiration below set PEEP) or breath-stacking caused by double triggering, tidal volume was progressively increased until a maximum of 8 mL/kg. If the latter did not solve the asynchrony, paralysis was reinstituted.

When the PEEP levels reached values ≤ 8 cmH_2_O with F_I_O_2_ ≤ 50% according to the PEEP-F_I_O_2_ table, patients could be ventilated in pressure support mode with pressure support of 5, 10, 15, or 20 cmH_2_O. Tolerance to pressure support was determined according to clinical criteria: no respiratory distress, respiratory rate ≤ 35 breaths/min, peripheral oxygen saturation ≥ 88%. If tolerance goals were met, pressure support level was decreased each 1–3 h until a minimum level of 5 cmH_2_O. Once the patient was transitioned to pressure support mode, patients were given control of their tidal volume with no limit imposed. If the patient did not tolerate the pressure support level, pressure support was increased at 5 cmH_2_O steps until the maximum value of 20 cmH_2_O. If this change failed to improve tolerance, patients were switched back to volume-controlled ventilation with a tidal volume of 6 mL/Kg.

### Extended protection group

In the intervention arm, patients underwent a second recruitment maneuver and PEEP titration, both equal to the maneuvers performed pre-randomization. The selected PEEP was defined as that which corresponded to equal amounts of collapse and overdistension estimated with the EIT. ΔP was adjusted to ≤ 15 cmH_2_O.

Strictly controlled ventilation was required for at least 48 h [19, 20]. In patients with PEEP > 15 cmH_2_O after the initial 48 h post-randomization, a new decremental PEEP titration starting at the current PEEP level was recommended if the ventral-to-global ratio of tidal ventilation was < 0.45 combined with persistently low respiratory system compliance (< 0.3 (mL/Kg)/cmH_2_O). After the first 48 h post-randomization, patients with PaO_2_:F_I_O_2_ ratios ≥ 150 mmHg and compliance of the respiratory system ≥ 0.3 (mL/Kg)/cmH_2_O were switched to pressure support ventilation. During the assisted phase, ΔP ≤ 15 cmH_2_O remained a target until extubation. With the goal to avoid or revert diaphragmatic atrophy, assisted ventilation at the lowest tolerated pressure support was encouraged whenever the lung was within the protective ΔP range. The estimation of the total driving pressure was obtained in two ways. First, by performing an end-inspiratory pause to allow time for the inspiratory muscles to relax and obtain stable plateau pressures [8, 21–23]. Driving pressure was then computed as the plateau pressure minus PEEP. Second, we performed an end-expiratory pause to estimate inspiratory pressure (occlusion pressure, Pocc). With this method, driving pressure was obtained as 0.75 × Pocc + pressure support as described previously [24, 25]. These maneuvers were performed at least once a day. When these two estimates were within 3 cmH_2_O from each other, we used their mean to indicate the patient driving pressure. Conversely, when the two estimates of driving pressure diverged by more than 3 cmH_2_O, we obtained driving pressure by dividing the observed tidal volume by the static respiratory system compliance measured with the patient paralyzed with short-acting neuromuscular blockers. This compliance measure obtained in the morning was considered valid for the following 24 h.

$${\Delta P}_{Total}=\frac{Tidal\, volume}{{Complaince}_{respiratory\, system}}$$

For example, a patient with a measured respiratory system compliance of 0.3 (mL/Kg)/cmH_2_O and with a tidal volume of 6 mL/Kg during assisted breathing would have an estimated ΔP of 20 cmH_2_O indicating that tidal volume should be lowered to 0.3 (mL/Kg)/cmH_2_O × 15 cmH_2_O = 4.5 mL/Kg.

Stepwise measures to try to lower ΔP were: increase PEEP by 2 cmH_2_O, increase F_I_O_2_ to obtain peripheral oxygen saturation > 95% [26, 27], administer or increase propofol dose [28], infuse sodium bicarbonate to target pH > 7.37 [29], and partial neuromuscular blockade [30, 31]. These measures were not mandated by the protocol; rather, they were advised as considerations for clinical judgment. The attending healthcare team retained the discretion to integrate other clinical variables into their decision-making process regarding the adoption of any suggested interventions. This flexible approach facilitated tailored patient care strategies.

### Primary outcome

The primary outcome was the modified Lung Injury Score (_m_LIS) assessed daily until day 28. Originally ranging from 0 to 4 points, the LIS is the average of four domains: PEEP, chest X-ray, PaO_2_/F_I_O_2_ ratio, and C_RS_. The score was modified in two ways: to accommodate hierarchically hard outcomes and to promote a more even comparison with a higher PEEP strategy. Thus, the modifications included (Additional file 1: Table S1): (1) patients who died before day 28 automatically received a score of 5; (2) patients extubated before day 28 and censored alive received a score of zero from the day of extubation onwards; (3) because post-randomization PEEP was nearly fixed in the EIT arm according to the protocol (except when a new titration was required—see Additional file 1), we used an F_I_O_2_-based score instead of a PEEP-based score for computation of the _m_LIS. This transformation allowed us to perceive improvements in lung function by progressive reductions of FIO_2_, instead of progressively lower PEEP levels. For the ARDSnet strategy, both variables are nearly equivalent for this purpose. The score was additionally calibrated between arms, to produce matched FiO_2_ scores immediately after randomization, despite different mean PEEP levels (for details, refer to the Additional file 1: Figure S1). The X-ray analysis was performed by four examiners blinded to the group assignment. Grades from 0 to 4 were assigned for each patient every day, indicating the number of quadrants involved (details in Additional file 1). This _m_LIS was chosen for its ability to capture longitudinal changes in lung function and recovery. Furthermore, the _m_LIS provided a measure with sufficient statistical power to detect intergroup differences, even with a relatively small sample size.

### Secondary outcomes

Pre-planned secondary outcomes included high-oxygen-dependence free days, defined as how many days patients took to remain in a sustained manner alive and with ≤ 1 L/min of oxygen supplementation until day 28; mechanical-ventilation free days, defined as how many days patients took to remain alive and without mechanical ventilation in a sustained manner after randomization until day 28. For these time-to-event variables, patients who died were assigned the worst possible outcome. For example, a patient who was extubated on day five and died on day 7 was considered intubated at 28 days. The incidence of shock (defined as the requirement for vasopressors) or barotrauma (pneumomediastinum, pneumothorax, or subcutaneous emphysema as diagnosed by the attending healthcare team); incidence of acute renal failure requiring renal replacement therapy; and 28 day survival.

### Additional exploratory (post-hoc) outcomes

We included two exploratory, post-hoc outcomes: time to room air and survival to 60 days (see Additional file 1).

### Statistical analysis

The sample size calculation was based on an anticipated mean difference between groups of 1 point in the _m_LIS [32]. With an α = 0.05 and power of 0.8, we estimated a sample size of 128 patients, 64 for each group. The study was interrupted early due to low rate of patient recruitment. Patients were analyzed according to their randomization group. Categorical variables were expressed as count and percentage, and continuous variables, as mean (standard deviation) or median (interquartile range) as appropriate. For longitudinal data, we estimated missing data using interpolation. For continuous variables with repeated measurements over time (including the primary outcome), we used either linear mixed models or nonlinear exponential models for comparisons between groups based on their Akaike Information Criterion. For example, for the primary outcome, we modelled the treatment response using a nonlinear exponential decay mixed model:

$$mLIS=a+k\times {e}^{-t/tau}$$

According to this formulation, _m_LIS starts at *(a* + *k)* at time zero and decays with time constant *tau* to the asymptote *a*. The time constant tau represents the rate of improvement of the _m_LIS. We built Kaplan–Meier curves to estimate 28-day survival and other time-to-event outcomes, and the differences between the two survival curves was tested with the logrank statistic. Time-to-event outcomes were also analyzed with Cox proportional hazards and reported as hazard ratios with 95% confidence intervals. All hypothesis tests were two-tailed with a significance level of 0.05 and performed using the R software (R Core Team, 2016, Vienna, Austria). We also analyzed the primary, secondary, and exploratory outcomes using Bayesian analysis [33]. The results from the Bayesian method are reported as posterior estimate with the corresponding 95% credible interval (CrI).

## Results

### Patients

**Fig. 1:**
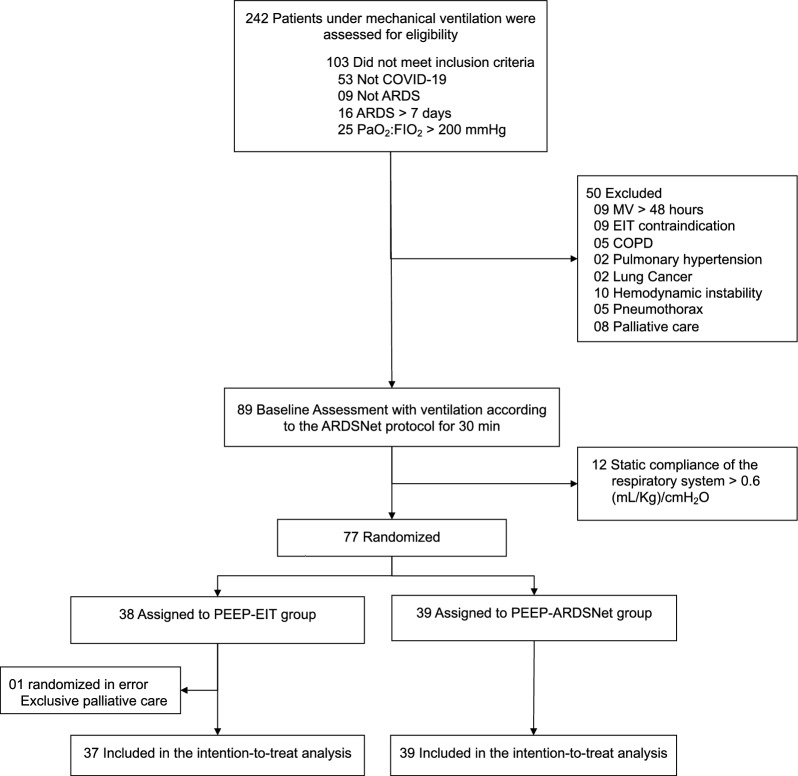
Consort flowchart

A total of 242 patients were mechanically ventilated from July 2020 to July 2022. From these, 31% fulfilled inclusion and exclusion criteria (Fig. 1). One patient under exclusive palliative care failed screening and was randomized in error. As a result, 76 patients were included in the intention-to-treat analysis, 37 in the extended protection group. Patients’ baseline characteristics were well balanced between groups (Table 1). The study was interrupted in March 2022, after observing three consecutive months without any new enrollment (Additional file 1: Fig. S2).

**Table 1:** Baseline characteristics

	ARDSNet	EIT
Variables	N = 39	N = 37
Age, years [mean (SD)]	61.5 (11.9)	60.9 (13.0)
Height, cm [mean (SD)]	166.6 (9.1)	167.4 (10.4)
Weight, Kg [mean (SD)]	84.6 (16.0)	89.8 (16.8)
Predicted body weight, Kg [mean (SD)]	61.1 (9.5)	62.0 (11.0)
Body mass index, Kg/m^2^ [mean (SD)]	30.6 (5.9)	32.1 (5.6)
SAPS3 [mean (SD)]	57.0 (11.2)	54.8 (10.0)
Probability of death—Latin America, % [mean (SD)]	40.2 (22.0)	36.9 (20.3)
Probability of death—Europe, % [mean (SD)]	30.8 (19.5)	27.7 (16.8)
PaO_2_/F_I_O_2_, mmHg (median [IQR])	102.2 (88.1 to 124.4)	102.4 (81.1 to 118.0)
pH (mean (SD))	7.33 (0.09)	7.35 (0.09)
Female, n (%)	15 (38.5)	13 (34.2)
Asthma, n (%)	2 (5.1)	0 (0.0)
Cancer, n (%)	0 (0)	0 (0)
Cardiovascular disease, n (%)	4 (10.3)	2 (5.3)
Chronic kidney disease, n (%)	0 (0)	0 (0)
COPD, n (%)	1 (2.6)	2 (5.3)
Diabetes, n (%)	17 (43.6)	13 (34.2)
Hypertension, n (%)	16 (41.0)	23 (60.5)
Smoking, n (%)	6 (15.4)	2 (5.3)
Dislipidemia, n (%)	0 (0.0)	2 (5.3)

### Primary outcome

**Fig. 2:**
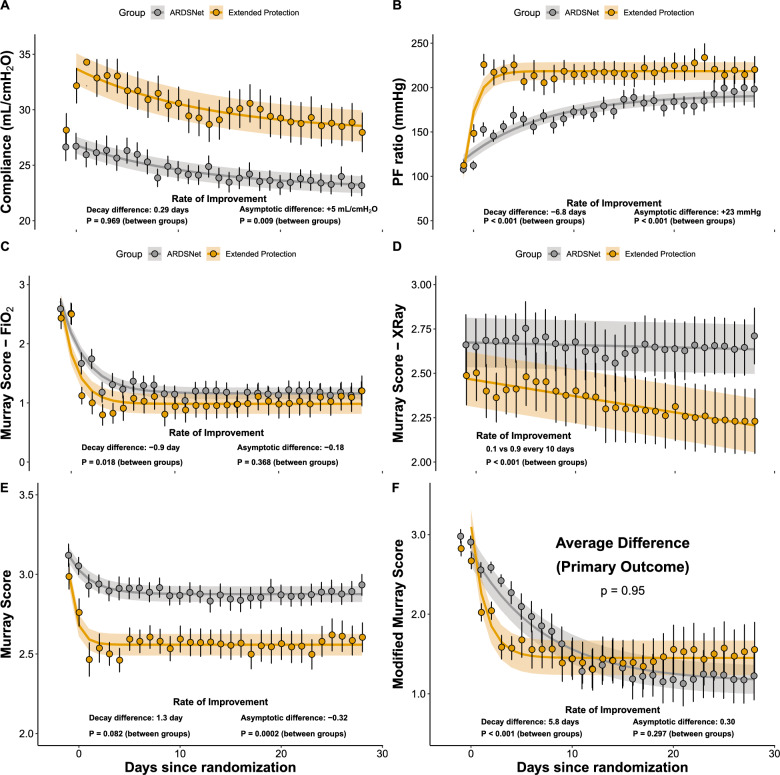
In panels **A**–**D**, the four components of the modified lung injury score (_m_LIS) are shown along the duration of the study. In panel **E** we show the average of the four components as in the original lung injury score publication. In panel **F**, the _m_LIS was hierarchically adjusted for survival and extubation rates. All variables were modeled using a mixed-model exponential fit except for the X-ray, which was modeled as a linear model with interaction with time. The intervention group is displayed in orange, and the control group, in gray. Markers indicate average values and error bars indicate standard error of the mean. The continuous line and shaded area represent modeled average predicted values with the respective standard error of the mean. Few missing data were interpolated along time within individuals with last observed non-missing data carried forward. Linear or nonlinear mixed models were used for repeated measurement comparisons between groups. The nonlinear models used exponential decay mixed models. For example, the primary outcome was modeled as: $mLIS=a+k\times {e}^{-t/tau}$. According to this formulation, _m_LIS starts at *(a* + *k)* at time zero and decays with time constant *tau* to the asymptote *a*. Differences in time-constant tau represents the rate of improvement (time to achieve 63% of the maximum improvement), analogous to interaction terms in the linear models. Differences in the asymptote mean that one group achieved higher maximum or final improvement, analogous to a significant between-subject effects

The average _m_LIS score was not different between groups (P = 0.95, primary outcome) (Table 2, Fig. 2). Of note, there was a faster decline in the _m_LIS in the extended protection group along the first week post-randomization (Table 2 and Fig. 2F, tau 7.23 vs. 1.41 days, P < 0.001). This difference was no longer visible by day 28 (P = 0.297).

**Table 2:** Primary, Secondary, and Exploratory Outcomes

	ARDSNet N = 39	EIT N = 37	Mean difference or relative risk (95% confidence interval)	P value	Posterior median difference or relative risk (95% credible interval)	Posterior probability that intervention is better (%)
Primary outcome						
mLIS	1.58 (1.19; 1.97)	1.56 (0.60; 2.53)	− 0.17 (− 0.59; 0.56)	0.95	− 0.01 (− 0.59; 0.56)	51
Rate of improvement (days)	7.2 (4.9; 9.6)	1.41 (0.91; 1.90)	− 5.82 (− 8.19; − 3.46)	< 0.001	− 5.59 (− 7.1; − 4.2)	100
Secondary outcomes						
Time to High-O_2_ Independence (days)	–*	-–*	2.25 (0.99; 5.10)	0.05	2.29 (1.03; 5.58)	98
Time to Extubation (days)	12 (10; 21)	12 (6; 28)	1.04 (0.61; 1.78)	0.88	1.03 (0.60; 1.73)	44
Shock (n, %)	25 (64.1)	25 (65.8)	1.03 (0.40; 2.65)	1.00	1.04 (0.40; 2.67)	47
Barotrauma (n, %)	2 (5.1)	3 (7.9)	1.63 (0.26; 10.37)	0.95	1.75 (0.26; 15.14)	30
Renal Replacement Therapy (n, %)	13 (33.3)	14 (36.8)	1.22 (0.48; 3.12)	0.86	1.22 (0.48; 3.22)	34
28-day Survival (days)	–*	–*	1.76 (0.57; 5.40)	0.32	1.77 (0.60; 5.76)	16
Exploratory outcomes						
Time to Room Air (days)	–*	–*	2.51 (1.16; 5.39)	0.02	2.51 (1.18; 5.55)	99
60-day Survival (days)	–*	–*	1.18 (0.54; 2.58)	0.68	1.17 (0.54; 2.66)	35

^*^Median time-to-event cannot be determined due to fewer than 50% of the study participants reaching the event

Two of the four domains of the _m_LIS presented an immediate and sustained improvement in the extended protection group: C_RS_ (P = 0.009) and PaO_2_/F_I_O_2_ ratio (P < 0.001; Fig. 2A, B). The X-ray component presented a progressive and significant improvement that extended beyond extubation and became progressively more evident towards day 28 in the intervention group (P < 0.001; Fig. 2D). Overall, there was a significant reduction in the Murray score in the intervention group over the 28 day period (P < 0.001; Fig. 2E), although this difference disappeared after adjusting for mortality (assigned a score of 5) and extubation (assigned a score of 0).

### Secondary (planned) outcomes

**Fig. 3:**
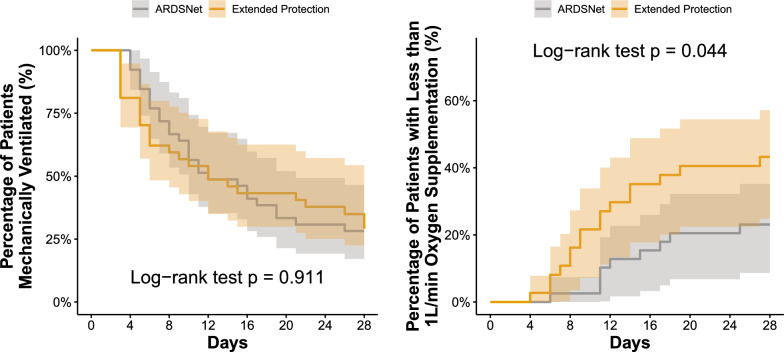
Time until liberation from the mechanical ventilation was similar between groups (**left**), but time until independence of high-oxygen supplementation was longer in the control group (**right**). The intervention group is displayed in orange, and the control group, in gray. High-oxygen supplementation was defined as the need of oxygen at flows ≥ 1 L/min. Time until breathing room-air, keeping SpO_2_ > 90% were also different and showed in the Additional file 1

Time to extubation (Table 2 and Fig. 3A) was statistically similar between groups (P = 0.884), but patients in the extended protection group had more high-oxygen-dependence free days until day 28 (P = 0.044, Table 2, and Fig. 3B).

**Fig. 4:**
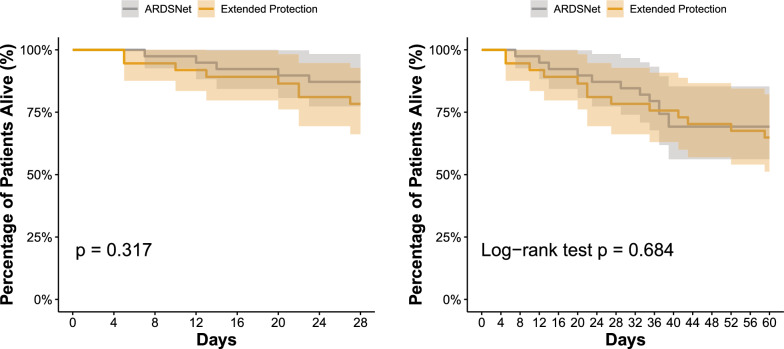
Kaplan Meirer Survival curves until 28 days (**left**) and 60 days (**right**). The intervention group is displayed in orange, and the control group, in gray. The shaded areas indicate 95% confidence intervals. Differences in survival were compared by logrank statistic

28-day survival was similar in both arms (P = 0.317, Table 2, and Fig. 4A). Other secondary outcomes (mechanical ventilation-free days until day 28; incidence of shock or barotrauma; incidence of acute renal failure requiring renal replacement therapy) were also similar (Tables 2 and 3).

**Table 3:** Other Outcomes

	ARDSNet	EIT	P value
Variables	N = 39	N = 37	
Ventilator-associated pneumonia, n (%)	7 (17.9)	5 (13.5)	0.830
Urinary tract infection, n (%)	2 (5.1)	2 (5.4)	1
Blood-stream infection, n (%)	1 (2.6)	2 (5.4)	0.963
Neuromuscular blockers, n (%)	37 (94.9)	35 (94.6)	1
Nitric oxide, n (%)	0	0	–
Prone positioning, n (%)	35 (89.7)	26 (70.0)	0.045
Number of prone sessions, median [IQR]	2 (1 to 3)	1 (0 to 3)	0.306
Extracorporeal oxygenation, n (%)	1 (2.6)	0	1
Ventilator-free days at Day 28, median [IQR}	14 (0 to 21.5)	11 (0 to 24.0)	0.784
Reintubation before day 28 (%)	3 (7.7)	6 (16.2)	0.476
Death until 28 days, n (%)	6 (15.4)	8 (21.1)	0.727
Death until 60 days, n (%)	12 (30.7)	13 (35.1)	0.872

### Physiological and respiratory variables

**Fig. 5:**
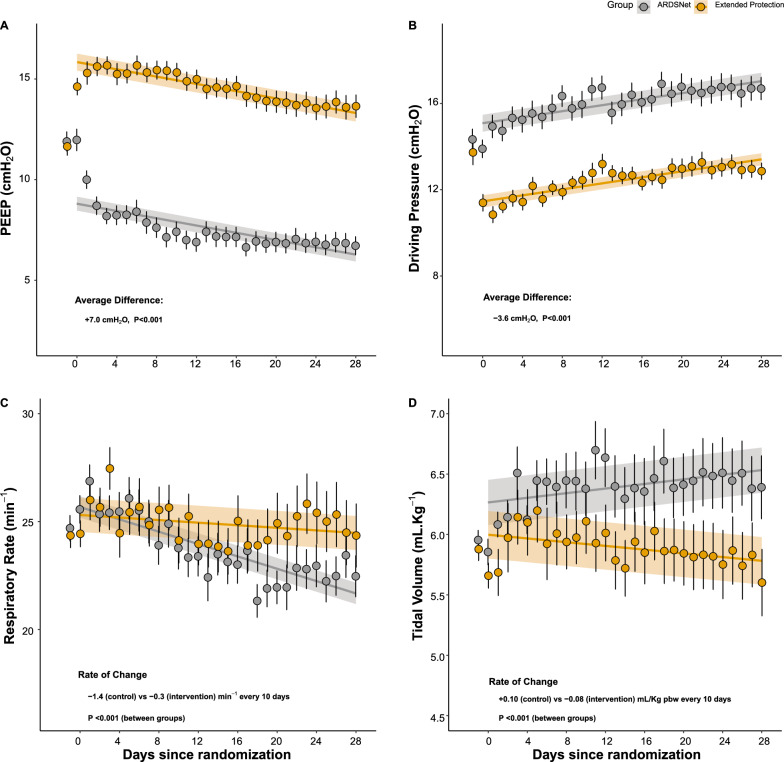
Positive end-expiratory pressure (**A**), driving pressure (**B**), respiratory rate (**C**), and tidal volume (**D**) over the 28 days of the study. The intervention group is displayed in orange, and the control group, in gray. Markers indicate average values and error bars indicate standard error of the mean. Missing data handling and statistics using mixed linear or non-linear models were performed as in Fig. 2. The upper two panels revealed significant differences for between-subjects factors, indicating a significant sustained difference from day zero (after randomization) till day 28. The lower two panels revealed significant differences only for the interaction factor, indicating similar values at day zero, but significantly higher differences along the time till day 28

Average PEEP was 7.0 ± 0.6 cmH_2_O higher (Fig. 5A), and average ΔP was 3.6 ± 0.5 cmH_2_O lower (Fig. 5B) in the extended protection group during the 28 days follow-up period. On average, protocol-defined protective values of ΔP (≤ 15 cmH_2_O) were guaranteed 88% of the time (Additional file 1: Figure S4), as compared to approximately 48% in controls.

PEEP levels suggested by the pre-randomization ARDSnet titration or EIT titration (both tested for all patients) were substantially different: PEEP_EIT_ was higher than PEEP_ARDSnet_ in 45 patients (59%), equal in 12 (16%) and lower in 19 patients (25%) (Additional file 1: Fig. S5).

Respiratory rate decreased faster in the control group (Fig. 5C), whereas tidal volume decreased in the intervention group but increased in the control group (P < 0.001 for the interaction; Fig. 4D). PaCO_2_ was higher in the intervention group, although not statistically different from controls (Additional file 1: Fig. S6). Arterial pH (Additional file 1: Fig. S7) and ventilatory ratio (Additional file 1: Fig. S8) were similar between groups.

Mean arterial pressure and heart rate were also similar (Additional file 1: Figures S9, S10). Likewise, daily creatinine levels (Additional file 1: Fig. S11), dialysis requirement (Table 2), and daily Sofa (Additional file 1: Fig. S12) scores were similar, even when considering non-pulmonary organ dysfunction (Additional file 1: Fig. S13).

### Exploratory Secondary outcomes

Time to room air was significantly shorter in the extended protection group (p = 0.02, Table 2 and Additional file 1: Fig. S15). Survival until 60 days was similar between groups (P = 0.684, Table 2, and Fig. 4B).

### Safety outcomes

Relevant safety outcomes are shown in Table 3, with no differences between groups. Norepinephrine requirements were similar between groups (Additional file 1: Fig. S16). Regarding sedative and paralytic agents: the intervention group used more propofol (Additional file 1: Fig. S17), whereas controls used more fentanyl (Additional file 1: Fig. S18). The use of prone positioning was less frequent in the intervention arm, but the use of neuromuscular blockade was equally frequent (Table 2). No differences in mean daily dose were found for the following drugs: midazolam, ketamine, cisatracurium, rocuronium (Additional file 1: Figs. S19–S21). Three accidental deaths in the intervention arm seemed non-related to the ventilatory strategy. Two fatal arrythmias were caused by severe hyperkalemia associated with impossibility of dialysis (peak of pandemic), and one accidental extubation (followed by irreversible cardiac arrest) also happened during the peak of pandemic.

## Discussion

In this pilot randomized study of extended lung protection vs. standard of care in patients with moderate to severe ARDS plus moderate to severely deranged mechanics of the respiratory system, we failed to find average differences between groups in the modified _m_LIS after 28 days, which was hierarchically adjusted for survival and extubation rates. However, even with adjustments for hard outcomes, the intervention group showed a faster rate of improvement of the _m_LIS-score (1.4 vs. 7.2 days to reach 63% of maximum improvement; P < 0.001), driven not only by oxygenation parameters, but also by a sustained improvement of X-ray (P = 0.001) and C_RS_ until day 28 (P < 0.001), a follow-up time when > 50% of patients were already extubated. The intervention group also achieved room air breathing and independence from high oxygen supplementation in a significantly shorter time. Other secondary outcomes like 28-day survival or time to extubation were not different between groups. Additionally, we showed that the extended lung protection protocol was feasible, reaching protective values of ΔP during 88% of the protocol duration (Additional file 1: Figure S4), as compared to 48% in the control group (a mean difference in ΔP of 3.6 cmH_2_O between groups throughout the protocol period).

We observed a significant difference in the LIS favoring the intervention group resulting from improvements in oxygenation, respiratory system compliance, and X-ray assessments. However, this difference was no longer evident when evaluating the modified version of the LIS, which was our primary outcome. The original LIS comprises four components directly linked to lung function: PEEP, respiratory system compliance, oxygenation (PaO_2_/FIO_2_), and radiographic appearance [32]. Our rationale for modifying the LIS was to adjust this physiological score to prioritize hard outcomes, such as extubation and mortality. This adjustment aimed to preclude the endorsement of a treatment approach that despite promoting better lung aeration, mechanics, and oxygenation might delay extubation or, even worse, increase mortality. Consequently, we decided to modify the originally proposed LIS assigning a score of 5 in the event of death and a score of 0 upon patient extubation. This modification made the effect in the LIS disappear because of a nonsignificant higher incidence of deaths and reintubation in the intervention group (reintubation was considered as “non extubated”, even if > 48 h reintubation).

**Fig. 6:**
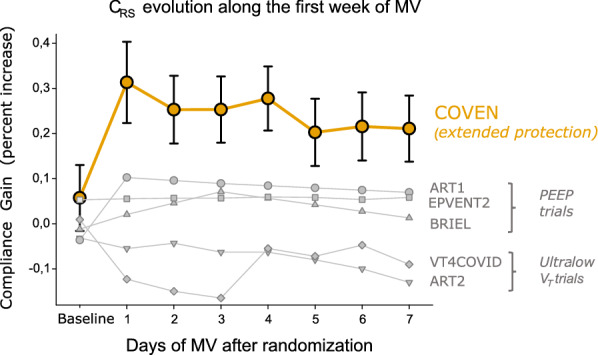
Evolution of compliance (of respiratory system—C_RS_) along the first 7 days of mechanical ventilation. Comparative results of recent trials on lung protection, including the current trial. Each line and respective symbol represent the mean differences observed in C_RS_ when comparing intervention versus the control arm within each trial. Results were calculated based on public data available in the original manuscripts, supplements or congress presentations. The standard error of mean could be only calculated for the current trial based on individual patient data. As shown, despite the eventual use of ultra-low V_T_ in patients of the intervention arm, the current COVEN trial presented a consistent improvement in C_RS_, exceeding 20% of initial values after one week of mechanical ventilation. Of note, other trials that tested ultra-low V_T_ not accompanied by high PEEP strategies presented deterioration of C_RS_ along the first week of mechanical ventilation. References used: ART-1 [35]; EPVENT2 [36, 51]; Briel et al., VT4COVID, and ART2 [34]

Compared to previous trials on lung protective strategies including high-PEEP and ultra-low-V_T_ trials [5, 34–37], our trial showed a notable sustained improvement in C_RS_ (Fig. 6), which is directly linked to the improvement of lung function over time. The magnitude of the reduction in ΔP associated to a sustained improved compliance along 2–4 weeks of mechanical ventilation is thus unprecedented in the era of lower tidal-volume strategies [2, 5, 34–37]. Interestingly, this was only partly achieved through opposite trends in tidal volume over time, which tended to increase in the control group and decrease in the intervention group. The stricter control of tidal volume in the intervention group came at the expense of a slower decrease in respiratory rate over time. However, based on the previous literature assessing the tradeoff between decreases in ΔP vs decreases in respiratory rate [38], we believe that our choice was justified. A larger portion of the ΔP effect size was likely attributed to gentler ventilatory settings, including a PEEP strategy that resulted in a better lung condition and optimized compliance. This choice of PEEP selection, as opposed to that based on oxygenation, has recently been shown to confer the best impact on patient effort increasing the odds of attaining total driving pressures compatible with lung protection [10, 12]. The maintenance of PEEP levels despite improvement in oxygenation or in X-rays is justified based on knowledge that even patients with normal lungs need substantial levels of PEEP during anesthesia in order to avoid postoperative collapse [39]. Based on this rationale, the triggers for PEEP reduction were not the traditional improvements in oxygenation, but clear signals of overdistension on EIT (ventral to global ratio of tidal ventilation < 0.45). During weaning, patients were submitted to a spontaneous breathing trial under relatively high CPAP levels of 8–15 cmH_2_O, before direct extubation to NIV—when equivalent CPAP levels were kept for 24–48 h. This approach has been recently tested by our group in a successful trial [40].

The choice of PEEP based on EIT, especially the crossing point between collapse and overdistension, is gaining interest in the literature because of its compromised solution with potential clinical benefit [11, 39, 41–45]. First, it usually coincides with a slightly positive (0–2 cmH_2_O) transpulmonary pressure, avoiding excessive lung collapse and hypoxemia even in super-obese patients [46]. Second, this PEEP selection commonly results in improved lung compliance, allowing a substantial reduction in ΔP [39]. And third, even when it indicates the need of high PEEPs (> 16 cmH_2_O), the hemodynamic tolerance is usually good because in such patients pleural and abdominal pressures tend to be elevated, a condition that maintains venous return and avoids increased afterload to the right ventricle [46–48]. Consistently, despite the higher median PEEP, the extended protection group presented good hemodynamic profile comparable to the traditional low-PEEP/F_I_O_2_ table group when considering arterial blood pressure, heart rate, renal function, SOFA scores, and need of vasopressors. Of note, this PEEP level was not necessarily higher than that selected according to the low-PEEP-F_I_O_2_ table (Figure e5). In 41% of the patients, this level was equal or lower.

Among the remaining strategies we used to control the ventilatory drive in the intervention arm, we sequentially applied: bicarbonate infusion targeting an arterial pH > 7.37 [29], higher use of propofol [28], lower use of opioids [49], peripheral oxygen levels closer to 97% (rather than 90–95%) [26, 27] and partial paralysis [30, 31] up to 7–10 days. This attempt to control the ventilatory drive with the goal of extending lung protection to the assisted phase is the main differential of our protocol compared with previous uses of electrical impedance tomography in patients with ARDS [41, 44]. We did not observe any case of profound weakness of respiratory muscles impairing the weaning, but we did not measure the impact of this strategy on peripheral muscles. A non-significant higher rate of reintubation in the intervention group could be potentially related to the use of prolonged partial paralysis but could also represent excessive enthusiasm during spontaneous breathing trials, after observing a fast improvement in lung function. The ideal weaning strategy after extended lung protection needs further study.

Our study has several limitations. First, it was a small, single-center pilot study, which limits the generalizability of our results. Second, we used a surrogate primary outcome, a modification of the lung injury score. Third, while our study focused on patients with ARDS due to COVID-19, we believe that our results may also apply to patients with ARDS due to other etiologies, as our protocol targeted the syndromic aspects of ARDS rather than the specific etiology. Fourth, our study may have inherent biases related to early stopping [50], especially the possibility of effect size magnification. Fifth, the intervention group exhibited enhanced oxygenation, thus necessitating less frequent application of prone positioning—a therapy with established clinical benefits. This disparity raises the possibility of an inadvertent bias against the intervention arm in the study outcomes. Finally, we tested a bundle of protection comprised of PEEP selection based on EIT and extended control of total ΔP during the assisted phase of mechanical ventilation. It is thus impossible to discern which component of the bundle was responsible for the differences between groups.

## Conclusion

The extended lung protection with strict control of ΔP for moderate to severe ARDS plus deranged mechanics is feasible and could be implemented in this pilot study with some beneficial impact on the recovery of lung function. Due to the early stopping of this study, we were not powered to assess the superiority of this strategy over the conventional low-PEEP/F_I_O_2_ ARDSnet strategy. Nevertheless, some promising and positive secondary outcomes encourage the test of such protocol in a larger trial—or at least the implementation of parts of it in new studies. We did not observe any evident side effect of the proposed strategy.

**Table:** 

**At a Glance Commentary** Scientific Knowledge on the Subject: The importance of early protective ventilation strategies following ARDS diagnosis is well-established. These strategies involve application of low tidal volumes and driving pressures, limited plateau pressures and respiratory rates as well as prone positioning. However, the optimal duration of this lung protection has not been studied
**What this study adds to the field:** In this Phase II Randomized Controlled Trial, we assessed the feasibility of an extended lung protection strategy through daily monitoring of driving pressures until extubation or the end of the 28-day follow-up. We maintained low driving pressures (≤ 15 cmH2O) throughout the mechanical ventilation period, a clear departure from the standard of care ARDSNet strategy, according to which low tidal volumes are only guaranteed until oxygenation meets the threshold for transitioning to pressure support modes. While the primary outcome showed a neutral impact on lung injury scores, the intervention arm exhibited an accelerated rate of recovery, leading to a shorter duration of oxygen dependence. This finding suggests that extended lung protection may have enduring positive effects on lung healing

### Supplementary Information

**Additional file 1.** Electronic online supplement.

## Data Availability

The datasets used and analyzed during the current study are available from the corresponding author on reasonable request.

## Abbreviations

ARDS: Acute respiratory distress syndrome
BMI: Body-mass index
ΔP: Airway driving pressure
EIT: Electrical impedance tomography
F_I_O_2_: Inspired fraction of oxygen
ICU: Intensive care unit
_m_LIS: Modified lung injury score
PBW: Predicted body weight
PEEP: Positive end-expiratory pressure
