# Supplementary material for: Impact of extended lung protection during mechanical ventilation on lung recovery in patients with COVID-19 ARDS: a phase II randomized controlled trial
Source: Ann Intensive Care. 2024 Jun 8;14:85. doi: 10.1186/s13613-024-01297-z (PMC11161454; doi:10.1186/s13613-024-01297-z)
Supplement: Supplementary file 1 — Additional file 1. Electronic online supplement. [file 13613_2024_1297_MOESM1_ESM.docx]

Online Supplement

**Impact of Extended Lung Protection during Mechanical Ventilation on Lung Recovery in patients with COVID-19 ARDS:**

A Phase II Randomized Controlled Trial

Eduardo L.V. Costa, MD^1,2,3*^

Glasiele C. Alcala, PhD^1,3*^

Mauro Tucci, MD^3^

Ewan Goligher, MD^4,5,6^

Caio Morais, PhD^1,7^

Jose Dianti, MD^4,5,6^

Miyuki A.P. Nakamura, PhD^1^

Larissa B. de Oliveira, PhD^3^

Sérgio M. Pereira, MD^1,4,5^

Carlos Toufen Jr, MD^3^

Carmen B. Valente, MD^3,8^

Carlos R. R. Carvalho, MD^1,3^

Marcelo B. P. Amato, MD^1,3^

* Co-first authors.

Institutional affiliations:

1. Laboratório de Pneumologia LIM-09, Hospital das Clinicas HCFMUSP, Faculdade de Medicina, Universidade de Sao Paulo, Sao Paulo, SP, BR.
2. Research and Education Institute, Hospital Sírio-Libanes, Sao Paulo, Brazil
3. Divisao de Pneumologia, Instituto do Coracao, Hospital das Clinicas HCFMUSP, Faculdade de Medicina, Universidade de Sao Paulo, SP, Brasil Interdepartmental Division of Critical Care Medicine, University of Toronto, Toronto, Canada
4. Keenan Research Centre, Li Ka Shing Knowledge Institute, St. Michael’s Hospital, Toronto, Canada
5. Toronto General Hospital Research Institute, Toronto, Canada
6. Departamento de Fisioterapia, Universidade Federal de Pernambuco, Recife (PE) Brasil
7. Respiratory ICU, University of São Paulo Medical School and Adult ICU Albert Einstein Hospital, São Paulo, Brazil

Summary

[1.	Primary outcome	4](#_Toc155871425)

[2.	X-ray analysis	7](#_Toc155871426)

[3.	Exploratory outcomes (post-hoc)	7](#_Toc155871427)

[4.	Population – rationale for the entry criteria	7](#_Toc155871428)

[5.	Patient Monitoring and Pre-randomization Measurements	10](#_Toc155871429)

[Pre-randomization Mechanical Ventilation according to the ARDSNet protocol	10](#_Toc155871430)

[Pre-randomization Mechanical Ventilation based on EIT Findings	11](#_Toc155871431)

[6.	Lung protective ventilation during the first 12 to 48 hours of enrollment	12](#_Toc155871432)

[ARDSNet arm	12](#_Toc155871433)

[Extended Protection arm	12](#_Toc155871434)

[7.	Lung protective ventilation after the first 12 to 48 hours of enrollment	13](#_Toc155871435)

[Measurement of total driving pressure and inspiratory muscle pressure	13](#_Toc155871436)

[Extended Protection arm	13](#_Toc155871437)

[Why EIT PEEP was kept almost unaltered until weaning	14](#_Toc155871438)

[ARDSNet arm	16](#_Toc155871439)

[8.	Statistical details and Bayesian sensitivity analyses	17](#_Toc155871440)

[9.	Results	19](#_Toc155871441)

[Rate of protective ventilation	19](#_Toc155871442)

[Counterfactual PEEP	20](#_Toc155871443)

[Arterial partial pressure of CO_2_	21](#_Toc155871444)

[Arterial pH	22](#_Toc155871445)

[Ventilatory ratio	23](#_Toc155871446)

[Mean arterial pressure	24](#_Toc155871447)

[Heart rate	25](#_Toc155871448)

[Serum creatinine	26](#_Toc155871449)

[Sequential organ failure assessment (SOFA)	27](#_Toc155871450)

[Non-respiratory SOFA	28](#_Toc155871451)

[Time to breathing room air	29](#_Toc155871452)

[Sensitivity Analysis Excluding the First 13 patients cared for in the Surge ICU	30](#_Toc155871453)

[Vasopressors, sedoanalgesia, and neuromuscular blockade	37](#_Toc155871454)

[References	43](#_Toc155871455)

# 1. Primary outcome

We chose to use the lung injury score as our primary outcome. This score is composed of four criteria, which are closely related to lung function: PEEP, respiratory system compliance, arterial partial pressure of oxygen to inspired oxygen fraction (PaO_2_/FIO_2_), and radiographic appearance (1). Our motivation was to find an outcome that would directly translate a potential effect of the intervention and that would also provide us with enough power to find differences between arms with a limited sample size. Being a pilot, single-center study, it would not be feasible to pursue hard outcomes such as mortality. Conversely, we did not want our results to support the intervention if it potentially caused harm. For this reason, we decided to modify the originally proposed lung injury score attributing a score of 5 if the patient died and a score of 0 if the patient was extubated. Additionally, we decided to modify the PEEP criterion because randomization, an exogenous factor, and not severity of the underlying lung disease would dictate this criterion in the intervention arm, which resulted in a relatively stable PEEP throughout the ventilation period. We used data from the ALVEOLI trial (2) of lower vs. higher PEEP to transform the PEEP criterion into an F_I_O_2_ criterion. Our goal with this transformation was to obtain equivalent lung injury scores in both arms on the first day after randomization, before any relevant change to the lung function itself had occurred. The F_I_O_2_ choices were based on the low PEEP-F_I_O_2_ table for the control arm and on the high PEEP-F_I_O_2_ table for the intervention arm (Table S1).

| **Table S1** | | | | | |
| --- | --- | --- | --- | --- | --- |
|  |  |  |  |  |  |
| **Score** | **0** | **1** | **2** | **3** | **4** |
| **PaO_2_/F_I_O_2_ (mmHg)** | ≥ 300 | 225-299 | 175-224 | 100–174 | < 100 |
| **Chest X-ray** | normal | 1 point for each quadrant | | | |
| **F_I_O_2_**  (EIT arm) | <40% | 40-49% | 50-54% | 55-79% | ≥80% |
| **F_I_O_2_**  (ARDSNet arm) | <40% | 40-50% | 51-69% | 70-89% | ≥90% |
| **C_RS_ (mL/cmH_2_O)** | ≥ 80 | 60 – 79 | 40 – 59 | 20 – 39 | ≤ 19 |
| PaO_2_/F_I_O_2_: ratio between the arterial partial pressure of oxygen (PaO_2_) and the inspired fraction of oxygen (F_I_O_2_); EIT: electrical impedance tomography; ARDSNet: acute respiratory distress syndrome network | | | | | |

This modified version of the lung injury score produced balanced scores between the two arms of the ALVEOLI trial (Figure S1) and was used to track changes in lung function by monitoring changes in F_I_O_2_, which were allowed in both arms.

Figure S1 – The Lung Injury Score (Murray score) applied as originally described to patients in the ALVEOLI trial on the first day post-randomizatioin (left) vs. the modified Lung Injury Score applied to the same patients (right). Note that the replacement of the PEEP criterion with a criterion based on the inspired fraction of oxygen created balance between groups.

# 2. X-ray analysis

The X-ray analysis, conducted to integrate the Murray score, was performed a posteriori by examiners blinded to the group assignment. Grades from 0 to 4 were assigned for each patient every day, indicating the number of quadrants involved. As additional quality control measures, four distinct physicians were tasked with grading the X-rays. Prior to beginning the evaluation process, each physician underwent specialized training to improve uniformity in grading. X-rays were performed on most days. Missing values were imputed by interpolation. We employed the weighted kappa statistic with squared weights (R Package: irr, version: 0.84.1) to evaluate the level of agreement among examiners in their evaluations of the X-ray assessments. In order to compute the mean weighted kappa along with 95% confidence intervals, we conducted 1000 iterative sampling iterations, with each iteration involving the selection of one sample per patient. This approach was chosen to effectively address the repeated nature of observations across various assessment days. The resultant kappa was 0.602 (CI 0255 – 0831).

# 3. Exploratory outcomes (post-hoc)

We included two exploratory, post-hoc outcomes: time to room air, survival to 60 days. Time to room air was a modification of the originally proposed high oxygen dependence free days. Time to event was defined as the interval from randomization to when the patient was able to breathe without any oxygen supplementation.

# 4. Population – rationale for the entry criteria

All patients under invasive mechanical ventilation, fulfilling the Berlin ARDS criteria (3) caused by SARS-COV2 (confirmed or with strong suspicion) were screened. The inclusion criteria were: PaO_2_/FIO_2_ ratio below 200 mmHg and respiratory system compliance (C_RS_) ≤ 0.6 mL/cmH_2_O/Kg of predicted body weight (PBW). We used this additional criterion based on respiratory system mechanics to enrich the trial population with patients with a higher likelihood to benefit from the intervention (4, 5). This compliance cutoff selected patients more likely to present high driving pressures using the current standard of care, thus more likely to benefit from further protection (5). According to this entry criterion, a patient with tidal volume of 6 mL/Kg would require a driving pressure of ≥10 cmH_2_O.

The exclusion criteria were age < 18 years, active bronchopleural fistula, history of chronic and disabling respiratory disease requiring home oxygen treatment, chronic pulmonary arterial hypertension, large intrathoracic tumoral mass, electrical impedance tomography monitoring contraindications (such as thoracic wounds or burns, electronic implantable devices), hemodynamic instability (systolic pressure < 80 mmHg or mean arterial pressure < 60 mmHg despite vasopressor drugs; and/or heart rate < 55bpm), patients at risk of intracranial hypertension or post cardiopulmonary resuscitation (first 72 hours), pregnancy, impossibility of monitoring with EIT (e.g., unavailability of machines), not commitment to full support or life expectation < 24 hours, refusal of the attending physician to include the patient in the study.

The study was interrupted prematurely because of low inclusion rate in March 2022 after including 76 patients, corresponding to 59% of the planned sample size (Figure S3). This decision was supported by the ethical committee, after declaration of the end of the pandemics.

Figure S2 – Study recruitment rate

# 5. Patient Monitoring and Pre-randomization Measurements

All patients were monitored with ECG, arterial invasive pressure, SpO_2_ (oxygen saturation by pulse oximetry), mainstream capnography and with electrical impedance tomography (EIT) (ENLIGHT ®1800 or 2100, Timpel, Brazil) as part of our ICU routine for moderate/severe ARDS. An electrode belt suitable for the chest perimeter of the patient was placed over the chest surface and a disposable flow/pressure transducer of the EIT device was connected to the proximal airway. We certified that all patients were under adequate sedation and neuromuscular blockade during baseline measurements. All patients were registered in a customized online platform for data entry (Redcap, Vanderbilt, USA).

## Pre-randomization Mechanical Ventilation according to the ARDSNet protocol

Initially, all patients were ventilated according ARDSNet low PEEP-F_I_O_2_ table protocol (6). Patients were ventilated in volume-controlled ventilation (VCV) with tidal volume adjusted to 6mL/Kg of predicted body weight (PBW) or lower to keep plateau pressure ≤ 30 cmH_2_O. When pH was below 7.15, tidal volume was increased in 1 mL/Kg or 2 mL/Kg, and plateau pressures above 30 cmH_2_O were allowed. Respiratory rate (RR) was initially adjusted to maintain the minute ventilation before study inclusion and did not exceed 35 breaths/min, using an inspiratory to expiratory ratio between 1:2 to 1:1. PEEP and F_I_O_2_ were adjusted according the lower PEEP/F_I_O_2_ table of the ARDSNET protocol. Adjustments were performed each 5-10 minutes to keep the SpO_2_ between 90-95%. We considered that a PEEP/F_I_O_2_ combination was stable when no further changes were needed for >10 minutes. We kept this ventilation for 15 minutes and an arterial blood gas (ABG) was drawn (“Baseline ventilation”) together with EIT and other physiological variables.

## Pre-randomization Mechanical Ventilation based on EIT Findings

After observing hemodynamic stability and mean arterial pressure ≥80 mmHg, a recruitment maneuver was performed under pressure-controlled ventilation (PCV) with delta pressure of 15 cmH_2_O, I:E ratio 1:1, RR kept between 20-30 bpm titrated to end-tidal CO_2_ values, and FiO_2_ of 1. PEEP was increased in steps of 5 cmH_2_O every 30 seconds, until a maximum PEEP of 30 cmH_2_O in patients with body mass index (BMI) < 35 Kg/m^2^. In patients with BMI >35 Kg/m^2^, one further step was added, with plateau pressures reaching 50 cmH_2_O. The recruitment maneuver was interrupted if any of the following occurred: mean arterial pressure persistently < 80 mmHg or fall > 15 mmHg, oxygen saturation persistently below 90%. Thirty seconds after the last PEEP step, PEEP was decreased to 24 cmH_2_O and the ventilator was set to VCV with tidal volume of 5mL/kg (PBW), RR to keep the same end-tidal CO_2_, and FiO_2_ of 1. With the PEEP titration tool (Enlight®, Brazil), collapse and overdistension were estimated at each 2cmH_2_O decremental PEEP step every 30 s until a minimum PEEP level of 4 cmH_2_O. At the end of the titration, the PEEP associated with equal amounts of collapse and overdistension was identified (EIT_PEEP_) (7).

# 6. Lung protective ventilation during the first 12 to 48 hours of enrollment

## ARDSNet arm

Patients were placed on volume-controlled mode with the tidal volume set to 6mL/Kg PBW, plateau pressure ≤ 30 cmH_2_O, and respiratory rate (RR) ≤ 35 breaths/min. The target pH was 7.30 – 7.45. If pH was between 7.15 and 7.30, RR was increased to reach a pH > 7.30 or PaCO_2_ < 25mmHg (max = 35 breaths/min).	If pH was < 7.15 and RR was at the maximum value allowed, tidal volume was increased even if plateau pressure exceeded 30 cmH_2_O. In this scenario, sodium bicarbonate infusion was also considered. PEEP and F_I_O_2_ were set according to the PEEP-F_I_O_2_ table. At least *12 hours* after the start of the protocol, every patient was screened for the commencement of the weaning phase. If the patient reached an F_I_O_2_ ≤ 40%, he or she could be transitioned to pressure support mode with pressure support.

## Extended Protection arm

The selected PEEP (PEEP_EIT_) was defined in a post-randomization PEEP titration maneuver performed after a recruitment maneuver. As in the previous maneuver, PEEP_EIT_ was that which corresponded to equal amounts of collapse and overdistension (crossing point). Driving pressure was adjusted to ≤15 cmH_2_O, and F_I_O_2_ was set to meet oxygenation targets. PEEP_EIT_ was kept throughout the mechanical ventilation period except when PEEP_EIT_ was >15 cmH_2_O, oxygenation had improved (PaO_2_/F_I_O_2_ > 150 mmHg) but respiratory system compliance was persistently low (<0.3 (mL/Kg)/cmH_2_O) after the *initial 48 hours* post-randomization. In this scenario, if the ventral to global ratio of tidal ventilation (measured with EIT) was <0.45(8), a new decremental PEEP titration starting at PEEP_EIT_ was recommended. Strict controlled ventilation was required for *at least 48 hours*.

# 7. Lung protective ventilation after the first 12 to 48 hours of enrollment

## Measurement of total driving pressure and inspiratory muscle pressure

In both arms, total driving pressure was estimated several times a day. The estimation of the total driving pressure was obtained in two ways. First, by performing an end-inspiratory pause to allow time for the inspiratory muscles to relax and obtain stable plateau pressures(9–12). Driving pressure was then computed as the plateau pressure minus PEEP. Second, we performed an end-expiratory pause to estimate inspiratory pressure (occlusion pressure, Pocc). With this method, driving pressure was obtained as 0.75 x Pocc + pressure support as described previously(13, 14). When these two estimates were within 3 cmH_2_O from each other, we used their mean to indicate the patient driving pressure. Conversely, when the two estimates of driving pressure diverged by more than 3 cmH_2_O, we obtained driving pressure by dividing the observed tidal volume by the static respiratory system compliance measured with the patient paralyzed with short-acting neuromuscular blockers. This compliance measure obtained in the morning was considered valid for the following 24 hours.

$${\Delta P}_{Total}=\frac{Tidal volume}{{Complaince}_{respiratory system}}$$

## Extended Protection arm

After the initial 48 hours of rest, the patient could start the assisted or spontaneous breathing at the discretion of the ICU team. As per the routine of the ICU, PSV mode was recommended if the patient had a stable ventilatory drive. During this assisted phase, the strategies applied to reduce total driving pressure if necessary (>15 cmH_2_O) were:

1. To reduce pressure support, sometimes even to zero if this resulted in lower total driving pressures and no patient discomfort;
2. To increase PEEP by 2-4 cmH_2_O above the set EIT-PEEP value to reduce the neuromechanical coupling of the diaphragm thus lower driving pressures and tidal volumes;
3. To increase F_I_O_2_ up to target SpO_2_ = 98% - provided that this maneuver caused a decrease in ventilator drive (assessed by P0.1 or by the end-expiratory);
4. To increase sedatives (mainly propofol) to reduce respiratory drive;
5. To infuse IV bicarbonate targeting a pH ≥7.37 in order to decrease ventilator drive, even when hypercapnia was present;
6. To restart neuromuscular blockers targeting partial paralysis (15, 16).

### Why EIT PEEP was kept almost unaltered until weaning

As soon as the COVID-19 pandemic started, many patients in our ICU were included in the RECRUIT trial(17). We then realized that their average PEEP levels defined by EIT did not change substantially along the days or weeks (consistent with the results of Gomers et al (18)), provided that patient positioning and weight were preserved. Additionally, we realized that they were similar to levels detected in a previous study in patients with normal lungs submitted to general anesthesia (19) (see figure S4). This new information led us to conclude that each patient had his “signature PEEP”, correlated with body-mass index, but not much related to the improvement in lung condition. Thus, the decision to decrease PEEP levels was driven by signals of progressive overdistension, and not by improvements of oxygenation.

Figure S4 – PEEP identified at the crossing point between collapse and overdistension during a decremental PEEP trial in patients during the intraoperative period (upper graph) or intubated due to COVID-19 (middle graph). The lower graph shows the two patient populations together. Note the similar PEEP range in the two populations and the positive correlation with body mass index.

## ARDSNet arm

After the initial 12 hours of lung protection, patients could be ventilated in volume assist-control mode. During this assisted phase, tidal volumes were controlled with the same targets of the controlled phase. In case of excessive flow starvation (airway pressures during inspiration below set PEEP) or breath-stacking caused by double triggering, tidal volume was progressively increased until a maximum of 8 mL/kg. If the latter did not solve the asynchrony, paralysis was reinstituted.

When the PEEP levels reached values ≤ 8cmH_2_O with F_I_O_2_ ≤ 50% according to the PEEP-F_I_O_2_ table, patients could be ventilated in pressure support mode with pressure support of 5, 10, 15, or 20 cmH_2_O. Tolerance to pressure support was determined according to clinical criteria: no respiratory distress, respiratory rate ≤35 breaths/min, peripheral oxygen saturation ≥ 88%. If tolerance goals were met, pressure support level was decreased each 1-3 hours until a minimum level of 5 cmH_2_O. Once the patient was transitioned to pressure support mode, patients were given control of their tidal volume with no limit imposed. If the patient did not tolerate the pressure support level, pressure support was increased at 5 cmH_2_O steps until the maximum value of 20 cmH_2_O. If this change failed to improve tolerance, patients were switched back to volume-controlled ventilation with a tidal volume of 6 mL/Kg.

# 8. Statistical details and Bayesian sensitivity analyses

For longitudinal data, we imputed missing data using interpolation. Linear or nonlinear mixed models were used for repeated measurement comparisons between groups based on visual inspection of the data. The nonlinear models used were exponential decay mixed models. For example, the primary outcome was modeled as:

$$mLIS=a+k\times e^{-t/tau}$$

According to this formulation, _m_LIS starts at *(a + k)* at time zero and decays with time constant *tau* to the asymptote *a*. The time constant tau represents the rate of improvement of the _m_LIS. We built Kaplan-Meier curves to estimate 28-day survival, and the differences between the two survival curves was tested with the logrank statistic. Time-to-event outcomes were also analyzed with Cox proportional hazards and reported as hazard ratios with 95% confidence intervals. Oxygen-free days were compared with proportional odds model.

In sensitivity analyses (Table 2), we used Bayesian linear mixed effects regression clustered by patient to evaluate the effect of the intervention on the average mLIS. The model was fitted using the normal distribution to derive a) effect estimates with their corresponding 95% credible intervals, and b) the posterior probability of an effect. Specifically, we used the 50^th^, 2.5^th^, and 97.5^th^ percentiles of the posterior distribution to derive the median and 95% credible intervals (CrI) for the difference in mLIS between groups, respectively. The posterior probability of an effect was estimated as the posterior probability of mLIS (intervention) < mLIS (control).

We also used nonlinear (exponential) mixed effects regression clustered by patient to assess the rate of improvement of the _m_LIS in the two groups using the following model:

$$mLIS=a+k\times e^{-t/tau}$$

According to this formulation, _m_LIS starts at *(a + k)* at time zero and decays with time constant *tau* to the asymptote *a*. The time constant tau represents the rate of improvement of the _m_LIS. We used the 50^th^, 2.5^th^, and 97.5^th^ percentiles of the posterior distribution to derive the median time constants for both groups. The posterior probability of an effect was estimated as the posterior probability of time constant (intervention) < time constant (control).

For the Bayesian analysis of the effect of the intervention on time to extubation, time to high-oxygen independence, 28-day survival, and 60-day survival, we fitted a Bayesian survival regression model using the cox family and the logit link. We used the 50^th^, 2.5^th^, and 97.5^th^ percentiles of the posterior distribution to derive the median and 95% CrI for the hazard ratio (HR), respectively. The posterior probability of an effect was estimated as the posterior probability of HR <1.

For the Bayesian sensitivity analysis of the effect of the intervention on the oxygen-free days, shock, barotrauma, and renal replacement therapy, we fitted a Bayesian regression model using the cumulative distribution and the logit link. The posterior probability of an effect was estimated as the posterior probability of OR <1.

All models were fitted using 4 chains and 8000 iterations (4000 iterations for the warm-up period). Convergence was assessed by visual inspection of trace plots and R-hat statistic.

We used minimally informative priors given the lack of data regarding the effect of the intervention on all outcomes analyzed. All Bayesian models were built using {brms} ([doi: 10.18637/jss.v080.i01](https://app.readcube.com/)).

# 9. Results

## Rate of protective ventilation

Protective ventilation as defined by the protocol as total driving pressure ≤ 15 cmH_2_O was feasible in the PEEP-EIT arm throughout the mechanical ventilation period (Figure S5).

Figure S5 – Rate of protective mechanical ventilation defined according to total driving pressure.

## Counterfactual PEEP

Figure S6 – Counterfactual PEEP levels. PEEP levels defined before randomization in all patients according to the PEEP titration method later applied in each group. Note that PEEP-EIT was not necessarily higher. If ventilated according to the PEEP-EIT, 45 patients (59%) would have received a higher PEEP, while 21 patients (41%) would have received PEEP values equal or lower to the PEEP-ARDSNet.

Figure S7 – Arterial partial pressure of CO_2_ (PaCO_2_) along the 28 days of the study. Markers indicate average values and error bars indicate standard error of the mean.

## Arterial partial pressure of CO_2_

Figure S8 – Arterial pH along the 28 days of the study. Markers indicate average values and error bars indicate standard error of the mean.

## Arterial pH

Figure S9 – Ventilatory ratio computed along the 28 days of the study. Ventilatory ratio was computed as [minute ventilation (mL/min) × PaCO_2_ (mmHg)]/(predicted body weight × 100 × 37.5) as previously described (20). Markers indicate average values and error bars indicate standard error of the mean.

## Ventilatory ratio

Figure S10 – Mean arterial pressures along the 28 days of the study. Markers indicate average values and error bars indicate standard error of the mean.

## Mean arterial pressure

Figure S11 – Heart rate along the 28 days of the study. Markers indicate average values and error bars indicate standard error of the mean.

## Heart rate

Figure S12 – Serum creatinine levels along the 28 days of the study. Markers indicate average values and error bars indicate standard error of the mean.

## Serum creatinine

Figure S13 – Sequential organ failure assessment (SOFA) values along the first 15 days of the study. Markers indicate average values and error bars indicate standard error of the mean.

## Sequential organ failure assessment (SOFA)

Figure S14 – Non-respiratory sequential organ failure assessment (SOFA) values during the first 15 days of the study. Markers indicate average values and error bars indicate standard error of the mean.

## Non-respiratory SOFA

## Time to breathing room air

Figure S15 – Time to breathing room air.

Figure S16– Average daily dose of norepinephrine at baseline and at days 1, 3, 5, 7, 10, and 15. Markers indicate average values and error bars indicate standard error of the mean.

## Vasopressors, sedoanalgesia, and neuromuscular blockade

Figure S17 – Average daily dose of propofol at baseline and at days 1, 3, 5, 7, 10, and 15. Markers indicate average values and error bars indicate standard error of the mean.

Figure S18 – Average daily dose of fentanyl at baseline and at days 1, 3, 5, 7, 10, and 15. Markers indicate average values and error bars indicate standard error of the mean.

Figure S19 - Average daily dose of midazolam at baseline and at days 1, 3, 5, 7, 10, and 15. Markers indicate average values and error bars indicate standard error of the mean.

Figure S20 - Average daily dose of ketamine at baseline and at days 1, 3, 5, 7, 10, and 15. Markers indicate average values and error bars indicate standard error of the mean.

Figure S21 - Average daily dose of cisatracurium at baseline and at days 1, 3, 5, 7, 10, and 15. Markers indicate average values and error bars indicate standard error of the mean.

## References

1.	Murray JF, Matthay MA, Luce JM, Flick MR. An expanded definition of the adult respiratory distress syndrome. *Am Rev Respir Dis* 1988;138:720–723.

2.	The National Heart, Lung and BIACTN. Higher versus Lower Positive End-Expiratory Pressures in Patients with the Acute Respiratory Distress Syndrome. *New England Journal of Medicine* 2004;351:327–336.

3.	Ranieri VM, Rubenfeld GD, Thompson BT, Ferguson ND, Caldwell E, Fan E, *et al.* Acute respiratory distress syndrome: the Berlin Definition. *JAMA* 2012;307:2526–2533.

4.	Zampieri FG, Costa EL, Iwashyna TJ, Carvalho CRR, Damiani LP, Taniguchi LU, *et al.* Heterogeneous effects of alveolar recruitment in acute respiratory distress syndrome: a machine learning reanalysis of the Alveolar Recruitment for Acute Respiratory Distress Syndrome Trial. *Br J Anaesth* 2019;doi:10.1016/j.bja.2019.02.026.

5.	Goligher EC, Costa ELV, Yarnell CJ, Brochard LJ, Stewart TE, Tomlinson G, *et al.* Effect of Lowering Vt on Mortality in Acute Respiratory Distress Syndrome Varies with Respiratory System Elastance. *Am J Respir Crit Care Med* 2021;203:1378–1385.

6.	Ventilation with Lower Tidal Volumes as Compared with Traditional Tidal Volumes for Acute Lung Injury and the Acute Respiratory Distress Syndrome. *New England Journal of Medicine* 2000;342:1301–1308.

7.	Pereira SM, Tucci MR, Morais CCA, Simões CM, Tonelotto BFF, Pompeo MS, *et al.* Individual Positive End-expiratory Pressure Settings Optimize Intraoperative Mechanical Ventilation and Reduce Postoperative Atelectasis. *Anesthesiology* 2018;129:1070–1081.

8.	Yoshida T, Piraino T, Lima CAS, Kavanagh BP, Amato MBP, Brochard L. Regional Ventilation Displayed by Electrical Impedance Tomography as an Incentive to Decrease Positive End-Expiratory Pressure. *Am J Respir Crit Care Med* 2019;200:933–937.

9.	Bellani G, Grassi A, Sosio S, Foti G. Plateau and driving pressure in the presence of spontaneous breathing. *Intensive Care Med* 2019;45:97–98.

10.	Bellani G, Grassi A, Sosio S, Gatti S, Kavanagh BP, Pesenti A, *et al.* Driving Pressure Is Associated with Outcome during Assisted Ventilation in Acute Respiratory Distress Syndrome. *Anesthesiology* 2019;131:594–604.

11.	Teggia-Droghi M, Grassi A, Rezoagli E, Pozzi M, Foti G, Patroniti N, *et al.* Comparison of two approaches to estimate driving pressure during assisted ventilation. *Am J Respir Crit Care Med* 2020;202:1595–1598.

12.	Bianchi I, Grassi A, Pham T, Telias I, Teggia Droghi M, Vieira F, *et al.* Reliability of plateau pressure during patient-triggered assisted ventilation. Analysis of a multicentre database. *J Crit Care* 2022;68:96–103.

13.	Telias I, Junhasavasdikul D, Rittayamai N, Piquilloud L, Chen L, Ferguson ND, *et al.* Airway occlusion pressure as an estimate of respiratory drive and inspiratory effort during assisted ventilation. *Am J Respir Crit Care Med* 2020;201:1086–1098.

14.	Bertoni M, Telias I, Urner M, Long M, Del Sorbo L, Fan E, *et al.* A novel non-invasive method to detect excessively high respiratory effort and dynamic transpulmonary driving pressure during mechanical ventilation. *Crit Care* 2019;23:1–10.

15.	Doorduin J, Nollet JL, Roesthuis LH, Van Hees HWH, Brochard LJ, Sinderby CA, *et al.* Partial Neuromuscular Blockade during Partial Ventilatory Support in Sedated Patients with High Tidal Volumes. *Am J Respir Crit Care Med* 2017;195:1033–1042.

16.	Dianti J, Fard S, Wong J, Chan TCY, Del Sorbo L, Fan E, *et al.* Strategies for lung- and diaphragm-protective ventilation in acute hypoxemic respiratory failure: a physiological trial. *Crit Care* 2022;26:.

17.	Jonkman AH, Alcala GC, Pavlovsky B, Roca O, Spadaro S, Scaramuzzo G, *et al.* Lung Recruitment Assessed by Electrical Impedance Tomography (RECRUIT): A Multicenter Study of COVID-19 Acute Respiratory Distress Syndrome. *Am J Respir Crit Care Med* 2023;208:25–38.

18.	Van Der Zee P, Somhorst P, Endeman H, Gommers D. Electrical Impedance Tomography for Positive End-Expiratory Pressure Titration in COVID-19-related Acute Respiratory Distress Syndrome. *Am J Respir Crit Care Med* 2020;202:280–284.

19.	Pereira SM, Tucci MR, Morais CCA, Simões CM, Tonelotto BFF, Pompeo MS, *et al.* Individual Positive End-expiratory Pressure Settings Optimize Intraoperative Mechanical Ventilation and Reduce Postoperative Atelectasis. *Anesthesiology* 2018;129:1070–1081.

20.	Sinha P, Calfee CS, Beitler JR, Soni N, Ho K, Matthay MA, *et al.* Physiologic analysis and clinical performance of the ventilatory ratio in acute respiratory distress syndrome. *Am J Respir Crit Care Med* 2019;199:333–341.
